# The Role of Nutraceutical Containing Polyphenols in Diabetes Prevention

**DOI:** 10.3390/metabo12020184

**Published:** 2022-02-17

**Authors:** Iva Fernandes, Joana Oliveira, Aryane Pinho, Eugenia Carvalho

**Affiliations:** 1 Laboratório Associado para a Química Verde—REQUIMTE, Departamento de Química e Bioquímica, Faculdade de Ciências, Universidade do Porto, Rua do Campo Alegre, 687, 4169-007 Porto, Portugal; iva.fernandes@fc.up.pt; 2 Center for Neuroscience and Cell Biology, Faculdade de Medicina, University of Coimbra, Rua Larga, Polo I, 1º Andar, 3004-504 Coimbra, Portugal; aryanepinho@cnc.uc.pt or; 3 Departamento de Ciências da Vida, Faculdade de Ciências e Tecnologia, University of Coimbra, Calçada Martim de Freitas, 3000-456 Coimbra, Portugal; 4 Instituto de Investigação Interdisciplinar, University of Coimbra, Casa Costa Alemão, Rua Dom Francisco de Lemos, 3030-789 Coimbra, Portugal; 5 APDP—Portuguese Diabetes Association, 1250-189 Lisbon, Portugal

**Keywords:** polyphenols, glucose, diabetes, insulin resistance, whole food, nutraceuticals

## Abstract

Research in pharmacological therapy has led to the availability of many antidiabetic agents. New recommendations for precision medicine and particularly precision nutrition may greatly contribute to the control and especially to the prevention of diabetes. This scenario greatly encourages the search for novel non-pharmaceutical molecules. In line with this, the daily and long-term consumption of diets rich in phenolic compounds, together with a healthy lifestyle, may have a protective role against the development of type 2 diabetes. In the framework of the described studies, there is clear evidence that the bio accessibility, bioavailability, and the gut microbiota are indeed affected by: the way phenolic compounds are consumed (acutely or chronically; as pure compounds, extracts, or in-side a whole meal) and the amount and the type of phenolic compounds (ex-tractable or non-extractable/macromolecular antioxidants, including non-bioavailable polyphenols and plant matrix complexed structures). In this review, we report possible effects of important, commonly consumed, phenolic-based nutraceuticals in pre-clinical and clinical diabetes studies. We highlight their mechanisms of action and their potential effects in health promotion. Translation of this nutraceutical-based approach still requires more and larger clinical trials for better elucidation of the mechanism of action toward clinical applications.

## 1. Introduction

### Diabetes

The diabetes epidemic is growing, especially in developing countries. It is fueled by increasing obesity across the lifespan, mostly due to physical inactivity and poor nutritional choices. Diabetes is estimated to reach 9.3% of the world’s population, in a short distant future [1]. Globally, over ~500 million people have type 2 diabetes (T2D) and another ~500 million have impaired glucose tolerance, in part due to poor nutrient intake [2]. In Portugal, with a Mediterranean-like diet that is increasingly changing to poor Westernized dietary habits, combined with decreasing physical activity, around 60% of the population has excess weight [3]. The T2D prevalence is one of the highest in Europe [4]. On the other hand, in India, for instance, where the population is largely vegetarian, relying on cereals for their diet, the obesity incidence is also steadily increasing. It is estimated that India will have approximately 80 million people with diabetes by 2030, leaving China (~45 million) and the USA (~35 million) behind by a significant margin [5]. The Indian population is considered “metabolically obese” in spite of being normal weight [6,7,8], thus at high risk for vascular disease and T2D development [9]. Therefore, diets based on simple sugars (sucrose/fructose) and poor in protein, particularly low on essential amino acids, as well as low in nutraceuticals, particularly phytochemicals, negatively affect metabolic and immune health [10,11].

Diabetes is characterized by hyperglycemia due to impaired insulin secretion and/or resistance to the peripheral action of insulin, in insulin-sensitive tissues, or both. Diabetes can be classified into the following major categories: type 1 diabetes (T1D), T2D, gestational diabetes (GD), and specific types of diabetes due to other causes, e.g., diseases of the exocrine pancreas (cystic fibrosis) or drug-induced diabetes (e.g., immunosuppressive agent used after organ transplantation) [12]. Chronic hyperglycemia in synergy with other metabolic alterations can cause damage to multiple organs, leading to the development of diseases and life-threatening health complications. Some of the most common complications of diabetes are cardiomyopathy, retinopathy, nephropathy, neuropathy, and foot ulcer development [13].

T2D is preceded by insulin resistance, one of the underlying causes of diabetes development [14,15,16,17,18]. Insulin resistance can begin years before symptoms are present, with high insulin levels even in the presence of normal glycemia, as demonstrated in pre-pubertal children with obesity [19]. Hypertension and heart disease are diagnosed at younger ages, as the age of T2D onset decreases [20,21]. This may lead to unhealthy aging and a significant decrease in the lifespan of the present younger generation [22]. People with insulin resistance and related co-morbidities are at higher risk of developing serious cardiometabolic complication, as well as severe complications caused by the recent severe acute respiratory syndrome coronavirus (SARS-CoV-2)/COVID-19 infection, in part due to their poor immune health [23,24,25]. Clearly, insulin resistance and diabetes have enormous societal and economic implications. It is vital to diagnose insulin resistance and diabetes at an early stage and identify high-risk groups to target for precision treatment, particularly precision nutrition.

Importantly, large clinical trials (i.e., ACCORD and ADVANCE) aimed at evaluating the impact of glucose lowering on diabetes complications have observed that lowering or normalizing glycemia does not equally lower or eliminate diabetes complications [26,27]. This suggests that glycemic control per se is not sufficient to prevent vascular and metabolic complications from developing. One of the possible hypotheses is that chronic exposure to high insulin levels, observed in insulin resistant subjects, even in a setting of normal glycemia is a major cause of inflammation, immune and metabolic dysregulation, increasing oxidative stress and inflammation, potentiating diabetes, and vascular complications [19,28].

With advances in pharmacological therapy, many antidiabetic agents have become available. Curiously, many pharmacological agents come from plant origin, including aspirin. In addition, new guidelines for precision medicine and precision nutrition to increase the health span seek to treat and control diabetes and its complications by increasing the patient life expectancy with quality. This approach greatly encourages the search for novel non-pharmaceutical molecules. This is in part due to their effect in combination with a healthy lifestyle (diet and exercise), which may be of great value for treatment and prevention of chronic diseases. Therefore, enriching the diet with plant-based functional foods and specific inflammation lowering phytochemicals from an early age may prevent or lower the obesity related chronic low-grade inflammation and may prevent diabetes from developing, which will lead to an increased life quality.

Present pharmacological agents, especially those used as multiple therapies to reduce hyperglycemia, may, in turn, also cause hypoglycemia, of potential risk for the patient [29]. The cost, potential side effects and benefits, glucose lowering efficacy, and dosing regimen are things to be taken into consideration before selecting a glucose-lowering or insulin-sensitizing medication. When oral drugs are not successful in modulating glucose and glycated hemoglobin (HbA1c) levels in people with insulin resistance or T2D, insulin can be utilized in monotherapy or together with various oral pharmacological agents. One of the limiting factors of insulin therapy is that it is mostly administered through injections. Despite showing favorable treatment effects, needle phobia causes poor compliance, which leads to inadequate glycemic control. Noninvasive alternatives would be to use commercially available inhaled or oral insulin instead. The oral absorption of insulin has already been described by the development of solid lipid nanoparticles (SLN) containing insulin, overcoming the side effects of the traditional route of administration [30]. Moreover, some of the major classes of oral antidiabetic medications available include biguanides, sulfonylureas, meglitinide, thiazolidinedione (TZD), dipeptidyl peptidase 4 (DPP-4) inhibitors, sodium-glucose cotransporter (SGLT2) inhibitors, and α-glucosidase inhibitors [31]. Besides the unquestionable change of the life quality of these patients, these drugs are not without related adverse side effects, such as hypoglycemia, gastrointestinal side effects, urinary tract infections, weight gain, and cardiovascular risk [31].

Since T2D is essentially a lifestyle problem, prevention by non-pharmacological interventions is not only important, but it has also become very promising. Since a sedentary lifestyle paired with excessive intake of poor-quality foods is the predominant cause of T2D development, lifestyle interventions are some of the safest and most natural ways of prevention. In addition, patients will be at a lower risk of experiencing serious secondary side effects by following non-pharmacological approaches. However, it must be noted that the effects of a non-pharmacological drug approach may not be everlasting, and treatment plan adherence is an important part to ensure the success of this approach, similarly to pharmacological approaches.

A healthy diet is the basis for the success of a life-changing pattern. Metabolic syndrome, an important global health problem, is characterized by a cluster of physiological abnormalities including hypertension, abdominal obesity, and insulin resistance, major risk factors for T2D development. In a meta-analysis of 50 original research studies (35 clinical trials, as well as 2 prospective and 13 cross-sectional studies), with over half a million participants, the effect of the Mediterranean diet on the metabolic syndrome as well as its associated conditions was assessed [32]. According to this extensive analysis, following a Mediterranean diet is associated with a significantly reduced risk of developing the metabolic syndrome or lowering its prevalence (log hazard ratio: −0.69, 95% confidence interval (CI): −1.24 to −1.16) [32]. The Mediterranean diet greatly impacted: the waist circumference (−0.42 cm, 95% CI: −0.82 to −0.02), high-density lipoprotein cholesterol (1.17 mg/dL, 95% CI: 0.38 to 1.96), triglycerides (−6.14 mg/dL, 95% CI: −10.35 to −1.93), systolic (−2.35 mm Hg, 95% CI: −3.51 to −1.18) and diastolic blood pressure (−1.58 mm Hg, 95% CI: −2.02 to −1.13), and glucose levels (−3.89 mg/dL, 95% CI:−5.84 to −1.95) [32].

Furthermore, greater adherence to this traditional dietary pattern is associated with the intake of phenolic compounds, a complex category of plant secondary metabolites. Moreover, it has been reported that long-term consumption of diets rich in phenolic compounds has a protective role against the development of T2D, due to their beneficial pharmacological activities [33].

## 2. Phytochemical Compounds: Structure, Food Sources, Bioavailability, and Metabolism

Phenolic compounds are secondary metabolites found in plants. They can be found in high amounts in our diet due to their presence in fruits, vegetables, cereals, chocolate, and beverages such as, coffee, tea, beer, cider, and wine [34,35,36,37,38,39]. These compounds are responsible for cellular growth and the regulation of fruit maturation. In addition, they are involved in the defense against pathogens as well as ultraviolet radiation [40]. This is, in part, due to their ability to react directly with substances in bacterial cells, preventing them from growing, as well as to have inhibitory capacity for some important enzymes. Phenolic compounds can also modulate protein regulation and capture or trap metals and substrates, thus interfering with bacterial growth [41,42].

Phenolic compounds may contribute to bitterness, astringency, color, and oxidative stability in food. Their daily intake can reach 900 mg/day in the general population, with values varying according to different dietary and lifestyle patterns [43]. For instance, the consumption of 20 g of dark chocolate can yield to an intake of more than 600 mg of phenolic compounds [38]. This is especially true for oligomeric and polymeric flavanols [44]. Countries where the population has a moderate consumption of coffee and tea can have a substantial increase in the phenolic compound intake by more than 250 mg and 40 mg per 100 mL, respectively [37,45]. Moreover, the inclusion of red wine in the diet can also yield the ingestion of more than 200 mg of phenolic compounds per cup, depending on the wine quality, age, grape variety, winemaking process, etc. [39]. However, the phenolic compound composition may not be the same. It may depend on its original natural source. Their bioavailability may not be the same. Different classes of these compounds may present different bioactivities.

**Figure 1:**
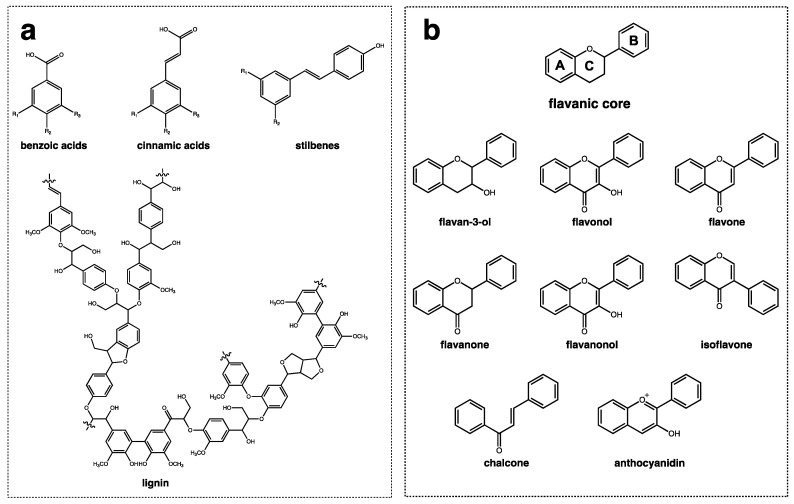
The general structure of phenolic compounds with (**a**) non-flavonoid compounds comprising benzoic, cinnamic acids, stilbenes, and lignins; and (**b**) flavonoid compounds represented by the flavanic core that is composed by two aromatic rings ((A) and (B)) and a pyranic ring (C) and their families of compounds: flavan-3-ol, flavonols, flavones, flavanones, flavanonols, isoflavones, chalcones, and anthocyanidins.

From a structural point of view, polyphenols are divided into two main groups, non-flavonoids, and flavonoids, as depicted in Figure 1. Non-flavonoids comprise different families of compounds with dissimilar structural features, including simple phenols, phenolic acids (benzoic and hydroxycinnamic acids), stilbenes (e.g., resveratrol), hydrolysable tannins, and lignans and polymeric lignins (Figure 1a). On the other hand, flavonoids are composed of a great diversity of families of compounds with a similar structural pattern, all of which have a flavanic core. The flavanic core is composed of two aromatic rings, A and B, that are linked through a heterocyclic pyranic ring C. The differences observed between each family of flavonoids is in the hydroxylation pattern of the rings and in the unsaturation degree of ring C [46]. Flavonoids comprise flavan-3-ols, flavonols, flavones, flavanones, flavanonols, isoflavones, chalcones, and anthocyanins (Figure 1b).

Phenolic acids, especially hydroxycinnamic acids and non-flavonoids, are one of the most abundant classes of phenolic compounds found in nature, and this includes *o*-, *m*-, and *p*-coumaric acid, sinapic acid, ferulic acid, and caffeic acid [47,48]. These phenolic compounds can be present as free carboxylic acids or as esters of tartaric and quinic acids, flavonoids, and carbohydrates [48]. Hydroxycinnamic acids are widely distributed in plant families, with several species being part of the diet or processed into beverages, including fruits, vegetables, and grains [48,49]. Regarding stilbenes, resveratrol is one of the most well-known compounds. It is produced by plants in response to injury or attack by pathogens such as bacteria or fungi. It plays an important role in the defense against stress-inducing conditions. Resveratrol has been found in some nutritional foods such as white squash, the roots of *Polygonum cuspidatum*, grapes, bilberries, blueberries, cranberries, and peanuts [50,51,52]. However, the amount of resveratrol present in these foods is generally low, ranging from 0.02 to 3 mg per 100 g (fresh weight) [53,54].

On the other hand, proanthocyanidins are reported as one of the major fraction of the total flavonoids ingested in Western diets [44]. These are polymers of flavan-3-ol widely present in vegetables, plant skins, seeds, flowers, fruits, and nuts [55]. Although results are scarce reporting the daily human consumption of these flavan-3-ol polymers, one study has described that the mean daily intake of proanthocyanidins, especially oligomers and polymers, in adults of the United States of America (USA)’s population was estimated to 57.7 mg/person [44]. On the other, the younger (2–5 year-old) and elderly (>60 year-old) populations were the groups with the highest consumption of proanthocyanidins, 68.2 mg/person and 70.8 mg/person, respectively, due to their higher ingestion of fruits and vegetables [44].

Anthocyanins are water-soluble pigments found in many fruits, flowers, and some legumes and are responsible for the red, violet, and blue colors found in nature. Structurally, they are characterized as glycosides of the flavylium cation polyhydroxylated and/or methoxylated. More than 500 anthocyanins have already been identified in nature, although cyanidin-3-*O*-glucoside represents around 50% of the anthocyanins present in red fruits [56]. Blackberries and blackcurrants contain up to 2–4 g per kg (fresh weight) of anthocyanins that are present mainly in the skin [56]. Red grapes can contain a similar content of anthocyanins (around 2.5 g per kg) and their processed beverages such as red wines, especially young wines, can contain up to 1 g per liter, with this amount varying according to the grape variety, the wine production methodology, the year of production, etc. [57,58].

Therefore, phenolic compounds, including flavonoids, namely proanthocyanidins and anthocyanins, and non-flavonoids such as phenolic acids and resveratrol, are a large and heterogeneous group of phytochemicals that are present in and are an important part of plant-based foods, such as tea, coffee, wine, cocoa, cereal grains, soy, fruits, and berries, which are part of our daily diet. Although there are no data regarding the amount of phenolic compounds needed to be ingested to have an healthy diet, the World Health Organization and Food and Agriculture Organization of the United Nations (WHO/FAO) report recommends a population-wide intake goal of 400 g of edible fruit and vegetables per day for the prevention of non-communicable diseases [59]. This amount of fruit and vegetables corresponds to a regular dietary intake of polyphenols of approximately 1–2 g per day [60].

Epidemiological evidence reporting on the ability of phenolic compounds to modulate or prevent T2D are scarce, and most date earlier than 2015 [33]. Generally, epidemiological studies show partial protection against the development of T2D due to the consumption of whole fruits for long periods of time. According to animal studies, the direct acute effect of (poly)phenols on postprandial glycemic response is relatively small, with a more modest effect on digestion and sugar absorption within the gut [61]. The notable effects from citrus (poly)phenol metabolites are on post-absorptive processes, such as the modulation of hepatic glucose metabolism and insulin sensitivity in target tissues [61].

On the other hand, and considering some of the most recent examples, there are some promising results to highlight. Diets naturally rich in phenolic compounds, from decaffeinated green tea, coffee, dark chocolate, blueberry jam, extra-virgin olive oil, and polyphenol-rich vegetables (artichokes, onions, spinach, and rocket), reduced plasma glucose, observed when measuring the total AUC (*p* = 0.038), likely by increasing early insulin secretion (AUC 0–30 min, *p* = 0.048) and insulin sensitivity in people at high cardiometabolic risk who had a large waist circumference [62].

In a double-blind, 8-week randomized controlled study involving 80 patients with diabetes, Brazilian green propolis (226.8 mg/day) rich in phenolic compounds was found to prevent worsening of blood uric acid and the estimated glomerular filtration rate [63]. In a controlled crossover trial, 11 healthy fasted volunteers consumed 300 mL of either light (LIR) or dark (DAR) roasted coffee, or water, followed by an oral glucose tolerance test (OGTT) [64]. Two coffees with dissimilar chlorogenic acid contents did not differentially affect glucose or insulin responses during an OGTT, but both raised insulin concentrations and reduced the insulin sensitivity index (ISI) compared with water, with no difference between them [64]. Daily consumption of polyphenol-rich extra-virgin olive oil improved metabolic control (significant reduction in fasting plasma glucose (*p* = 0.023), HbA1c (*p* = 0.039) levels, BMI (*p* = 0.012), and body weight (*p* = 0.012)), as well as the circulating inflammatory adipokine profile, in overweight patients with diabetes that were not being treated with insulin [65].

Guo and co-authors showed that risk of T2D development was decreased by 5%, with a 7.5 mg/day increment of dietary anthocyanin intake or with a 17 g/day increase in intake of blackberries, blueberries, raspberries, strawberries, or other berries [66]. For isoflavones, higher daidzein and genistein concentrations were associated with a lowered risk of diabetes in Korean and USA women, as well as in middle-aged and elderly Chinese volunteers [67]. In the framework of the described studies, there is clear evidence that the way phenolic compounds are consumed, their amount, and whether they are consumed acutely or chronically (short or long periods) does indeed affect their bioaccessibility, in turn also affecting their bioavailability. These are points of special relevance that deserve to be studied with more precision to drive research towards more accurate findings.

Special focus has recently been given to phenolic compounds that are easily extracted from plants with water or hydro-alcoholic/organic solvents, as well as to other factors influencing the bioavailability of free phenolic compounds [68]. Although the first mechanism proposed for the action of phenolic compounds in the human body is based on their direct antioxidant properties, these effects are no longer considered so relevant in vivo. The reason for this is that phenolic compounds are subjected to an intense metabolism due to the gastrointestinal digestion process. The native forms of these compounds present in food do not reach the target tissues in sufficiently high concentrations to have a significant effect in terms of neutralizing free radicals. Phenolic compounds are mostly like sugars, (glucose, galactose, rhamnose, xylose, rutinose, arabinopyranose, and arabinofuranose) O-conjugates at the C2 (chalcones), C3 (flavonols, anthocyanidins, and flavan-3-ols), or C7 (flavanones, flavones, and isoflavones) positions. These and other modifications (methylations and galloylation) add extra structural stability to phenolic compounds, reducing their ability to reach systemic circulation. Furthermore, their chemical structure determines whether these compounds are hydrolyzed and modified by enzymes before absorption, such as lactase phlorizin hydrolase (LPH) (at the enterocyte membrane) or *β*-glucosidase (CBG) (cytosolic, for polar glycosides). Their transport to liver via the portal vein by active, passive, or facilitated transportation can only occur after this metabolization process [69]. A major challenge associated with the bioactivity of phenolic compounds is their lack of bioavailability in vivo, which is primarily the result of coupled metabolic activities of conjugating enzymes and efflux transporters. Only 1–10% of the total phenolic compounds ingested are absorbed by the small intestine, largely due to the extensive metabolism they have to undergo [70]. A large portion of phenolic compounds are enzymatically transformed by methylation, glucuronidation, or sulfation in the liver, but also in the kidneys, intestine, and stomach [71]. Therefore, it is expected that these processes represent a significant barrier to the oral bioavailability of phenolic compounds and facilitates their biliary and urinary elimination by increasing their hydrophilicity. For example, anthocyanins are absorbed with poor efficiency in their parent forms and are therefore rapidly metabolized and eliminated [72]. Glucuronides and methyl conjugates of anthocyanins were identified in human urine and plasma by liquid chromatography coupled with mass spectrometry analysis after blackberry juice intake [73]. Furthermore, other metabolites were identified as degradation products, phenolic, hippuric, phenylacetic, and phenylpropenoic acids, which were present in the circulation for ≤48 h after ingestion [72].

In recent years, progress has been made on the identification of possible biochemical and molecular mechanisms of action of phenolic compound-derived metabolites, which depend fundamentally on their ingested doses through the food [43] and their effective bioavailability, or the amount that reaches the target tissue.

Importantly, phenolic compounds are used to slow starch digestion and in the transport of simple sugars across the intestinal epithelia, thereby reducing the plasma blood glucose spike after a meal. These effects are achieved through the inhibition of amylases, glucosidases, and glucose transporters present in the gastrointestinal tract and the brush boarder membranes [74]. The extent of inhibition by phenolic compounds is dependent on their molecular structure, intake doses, and the food matrix. For example, more polymerized procyanidins have more interaction sites with proteins and therefore will inhibit more efficiently α-amylase activity [75]. Likewise, Xiao and coworkers concluded that milk did not influence the oral relative bioavailability of pelargonidin anthocyanins under meal conditions. However, the oral relative bioavailability of pelargonidin anthocyanins was reduced by milk, by ~50% under fasting conditions (*p* < 0.05) [76]. Similarly, when these extracts of phenolic compounds are incorporated into food systems, they can interact with other biopolymers (carbohydrates, proteins, lipids) in the food matrix. This leads to lower inhibition of digestion and/or glucose transporters, compared to when they are ingested as an extract or in pure compound forms without additional matrix biomolecules, as often observed in most in vitro cell systems [77].

Greater consumption of specific whole fruits, particularly blueberries, grapes, and apples, has been significantly associated with a lower risk of T2D. On the other hand, increased consumption of fruit juice, instead of whole fruits (particularly from blueberries, grapes, and apples), seems to lead to an increased risk of T2D development, as observed in a study where 187,382 health professionals were free of major chronic diseases at baseline [78]. However, in a double-blind crossover acute trial in 34 healthy adult subjects, it was demonstrated that apple drinks enriched with phlorizin-rich apple extract reduced plasma glucose levels [79]. Another double-blind crossover trial with 25 healthy adult subjects showed that apple phenolic compounds reduced postprandial insulin, C-Peptide, and the insulin area under the curve at 30 min after a high-carbohydrate meal [80]. Recent findings suggest that the daily consumption of a cranberry beverage over 8 weeks may not have an impact on insulin sensitivity but it may be beneficial to lower triglycerides and change oxidative stress biomarkers including 8-isoprostane, a biomarker of lipid peroxidation and nitrate for nitric oxide production, in obese individuals with a proinflammatory state, with C-reactive protein levels > 4 mg/L [81].

Moreover, it is still not clear how the delivery of phenolic compounds in whole plant foods or the delivery of individual functional ingredients such as supplements impact the alteration of T2D risk markers. Few studies have been consistently performed to ascertain their functional specificity or their specific molecular effects. Random forms of consumption of these substances may be associated with the contradictory effects sometimes described. Considering the way these compounds are ingested, fruits are commonly consumed worldwide in both fresh and processed forms, especially as juices. While fruit consumption is perceived as beneficial for long-term health, the effects of fruit juices are more controversial and are associated with a high intrinsic sugar content. On the other hand, both forms are rich in bioactive compounds other than macro- and micronutrients. Furthermore, the bioaccessibility of phenolic compounds may importantly affect health outcomes.

**Figure 2:**
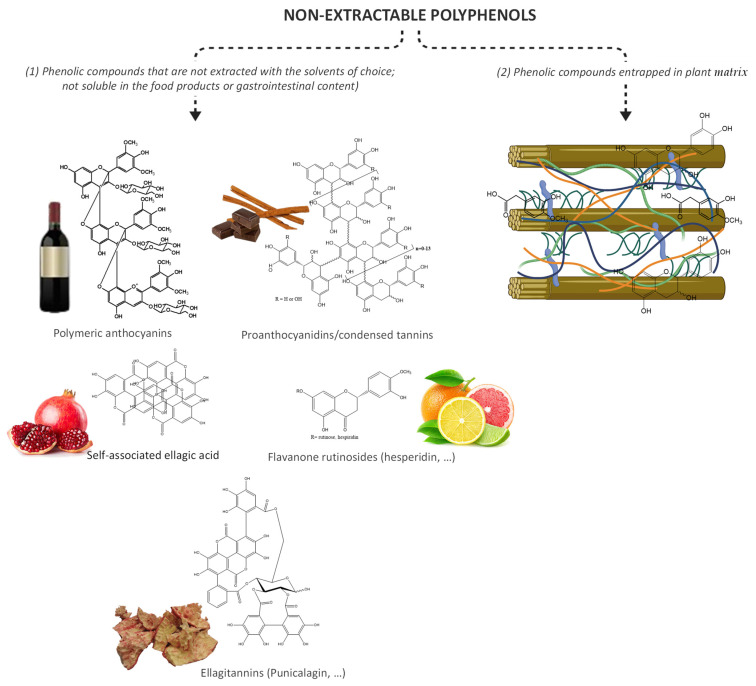
Non-extractable polyphenols, including (1) non-bioavailable polyphenols and (2) complex structures composed by cellulose microfibrils, hemicelluloses (xyloglucans, xylans (arabinoxylans, glucuronoxylan, and glucuronoarabinoxylan), glucomannans, and mixed-linkage glucans), pectins, lignins, bound polyphenols, and proteins.

Generally, phenolic compounds can be found in plant matrices in two forms, extractable and bound (Figure 2). Extractable phenolic compounds do not interact with other plant macromolecules and can be easily extracted after plant tissue disruption. On the other hand, bound phenolic compounds can be found cross-linked to cell wall macromolecules, such as cellulose, pectin, hemicellulose, lignin, and rod-shaped structural proteins [82] or can be physically entrapped within the food matrix [83]. In addition, the bound phenolic compounds also include all those phenolic compounds that are not extracted with water or mixtures with the most common organic solvents, such as ethanol, methanol, or acetone, and therefore are not assessed by the most used techniques for phenolic compounds analysis.

Hence, there has been considerable interest in understanding and exploiting the functions and health benefits of whole food systems, as these conjugates may have improved health attributes that may not be achieved using individual components, such as a great impact on microbiota modulation. For example, in a randomized, double-blinded, placebo-controlled, cross-over trial involving 22 healthy adult males, under daily supplementation of 500 mg of quercetin, no influence on the fasting glucose was observed [84]. However, the effectiveness of onion to ameliorate hyperglycemia and insulin resistance was observed in a parallel-design, randomized, triple-blind, controlled clinical trial, conducted on 56 eligible breast cancer patients (aged 30–63 years) [85]. In a meta-analysis, an increase in the consumption of fruit, especially berries, vegetables, and their fiber, was associated with a reduced risk of T2D development [86].

**Figure 3:**
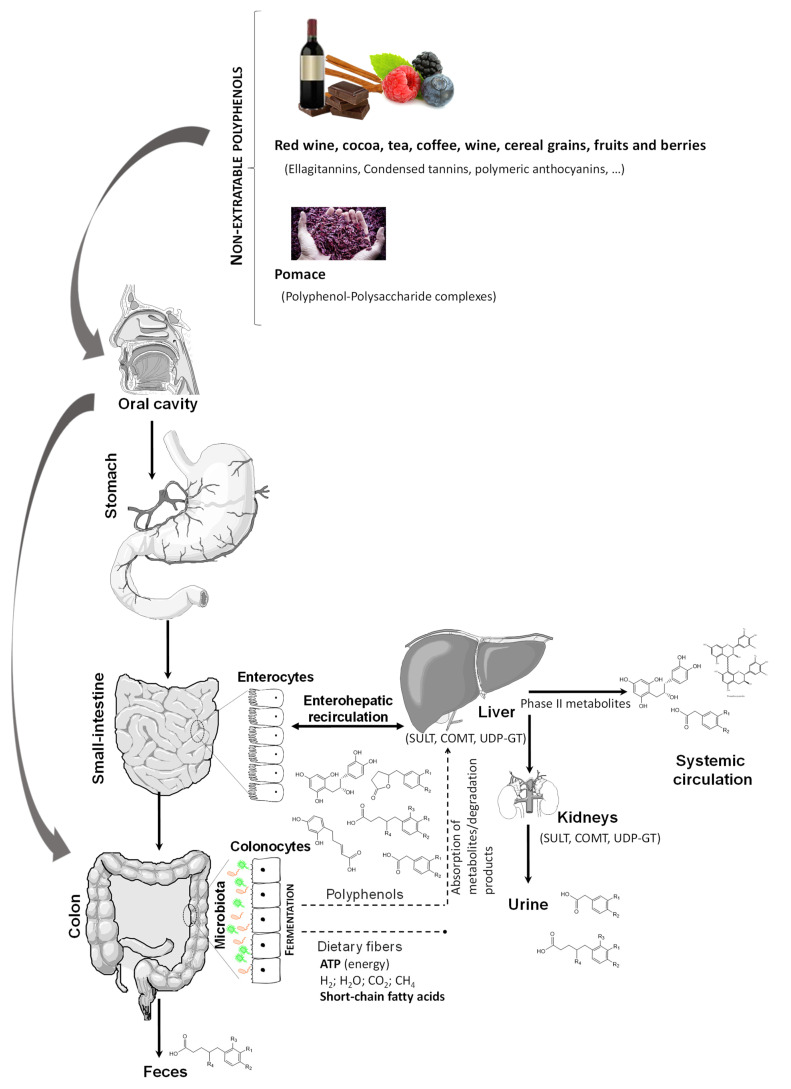
Oral ingestion and gut microbiota catabolism of non-extractable polyphenols. SULT: Sulfotransferases; UDP-GT: Uridine 5′-diphospho-glucuronosyltransferase; COMT: Catechol-*O*-methyltransferase.

Besides being a major part of dietary phenolic compounds, non-extractable phenolic compound complexes have been very poorly studied [87]. These complexes that are undigested in the upper digestive tract reach the colon, where the fermentation process is responsible for bacterial growth modulation [88] (Figure 3). Importantly, bacterial fermentation processes contribute to the release of free phenolic compounds, either as intact compounds or their derived metabolites (hydroxyphenylacetic and hydroxyphenylvaleric acids or urolithin, among others). These compounds can modulate microbiota inducing prebiotic-like effects on bacteria, while also being metabolized by intestinal bacteria into specific bioavailable metabolites [89]. Furthermore, there is evidence that intact parent (poly)phenols such as green tea catechins can inhibit glucose uptake in the gut. On the other hand, there is evidence that phenolic compound gut microbial metabolites, such as isovanillic acid 3-*O*-sulfate, a metabolite from the microbiome found in blood, primarily derived from consumed cyanidin-3-*O*-glucoside, could indeed stimulate glucose uptake in tissues by glucose transporter GLUT4- through PI3K-dependent mechanisms, as observed in the differentiated human skeletal muscle myoblast line, LHCN-M2 [90].

These different but complementary effects of parent compounds and microbial products are a promising line of research and could conceivably lead to synergistic effects between the parent compound and its microbial degradation products. However, it is important to keep in mind that changes in the gut microbiota composition and functionality among individuals may affect phenolic compound metabolism and therefore their health effects. For instance, gut dysbiosis induced by a high-fat (HF) diet can dramatically change the type and amount of endogenous and exogenous metabolites formed in the gut [91]. However, blackberry anthocyanin-rich extract can modulate gut microbiota composition and counteract some of the features of HF diet-induced dysbiosis.

Importantly, it should be highlighted that in vitro and perhaps also in vivo assays using pure phenolic compounds are probably physiologically irrelevant. The real environment, centered in a meal, which should be composed of a balanced amount of dietary phenolic compounds at physiological doses (between 1–10% of the ingested dose; if considering the Mediterranean diet, this should be around 1 g/day of total polyphenols), with included dietary fibers and other indigestible substrates [92]. Research supports the health benefits of a Mediterranean-style eating pattern that includes a “colorful” plate with several varieties of different foods, including various macro- and micro-nutrients. It is the combination of these foods that appear to confer most protection against disease, as the benefit is not as strongly observed when looking at single foods or pure nutrients [93].

Meanwhile, it should be highlighted that non-extractable phenolic compounds can have a fundamental role for nutritionists and physicians, since they are classified as macromolecular antioxidants [94]. Unlike the dietary antioxidants that are absorbed in the small intestine, macromolecular antioxidants reach the colon intact and, through the action of gut microbiota, may release to the colon simpler phenolic compounds. In combination, these antioxidants can help each other in promoting different antioxidant mechanisms. This synergy may support the need of a diet diversification to achieve the best health protection effects, as recommended by the Mediterranean diet.

Furthermore, phenolic compounds complexed with carbohydrates may act as a delivery nanostructure. These structures may protect the antioxidant from degradation and metabolism during gastro-intestinal passage and by enterohepatic recirculation ensuring the release at the bloodstream of “free” polyphenols. These “free” polyphenols (structures less polymerized or not conjugated with other types of molecules, such as sugars) suffer the action of gut microbiota, giving rise to metabolites that are much better absorbed reaching significant concentrations in plasma, which may persist for 3–4 days. Therefore, the intake of food sources rich in non-extractable phenolic compounds could be a strategy to reach a sustained systemic anti-inflammatory and antioxidant status in the human body thanks to the constant production of bioactive microbial metabolites [95]. This has been, so far, the first mechanism proposed for the action of phenolic compounds in the human body and their direct antioxidant properties, which, in the context of this health preventive picture, seem to be more than ever relevant.

In addition, recent findings suggest that some phytochemicals, such as sulforaphane, exhibit an hormetic effect in cells at low doses, activating signaling pathways that result in the increased expression of genes encoding cytoprotective proteins and antioxidant enzymes [96,97]. As such, the relatively small doses of phytochemicals ingested by humans are not toxic and instead induce mild cellular stress responses. The nuclear factor erythroid 2-related factor 2 (Nrf2)/antioxidant response element (ARE) pathway is an important cell signaling mechanism in maintaining redox homeostasis in humans [98,99,100]. The hormetic effect has, for instance, been reported for some flavonoids, including [101] luteolin, apigenin, quercetin, myricetin, rutin, naringenin, epicatechin, and genistein, and due to their antioxidant or prooxidant activity, depending on their concentrations, they contribute to redox homeostasis [98,102]. Furthermore, included in the category of “non-extractable polyphenols” are also the higher molecular weight phenolic compounds, polymeric structures, proanthocyanidins, and hydrolysable tannins. In addition, other phenolic compounds that are not complexes or oligomeric and that reach the colon almost unaltered since they are not soluble in food and in gastrointestinal content include hesperidin, due to solubility issues, and ellagic acid, as it self-associates into planar molecules, properties that greatly limit their absorption [95]. These points may explain, at least in part, the lack of evidence from clinical studies using phenolic compounds.

## 3. Intracellular Metabolites and Biomarkers Altered in Response to Nutraceutical Containing Polyphenol Intake

As discussed, for both prevention and management of diabetes, as well as its associated severe complications, glycemic control alone does not appear to be enough. Therefore, in recent years, there has been an increased focus on the identification of phenolic compounds in foods and in natural products. This is not only due to their high nutritional value, but also due to the more recent discovery of their significant health benefits, in part due to the presence of other bioactive components rather than the nutrients. This is especially true in the treatment of chronic disorders, including diabetes and its complications. In addition, there is a wide debate regarding the amount of bioactive components in phenolic compounds that are capable of exerting a protective health effect, as well as the right time and/or the duration of consumption (e.g., before or after a meal) [103]. In fact, bioavailability and bioaccessibility are important considerations in studies of bioactive compounds as functional dietary supplements. Furthermore, knowing that natural compounds have fewer side effects compared to pharmacological therapies, they are general accepted by the population, who consume them for health promotion or as adjuvants of traditional therapy [104].

At the moment, there is a wide commercialization of new classes of products based on enriched or concentrated mixtures of bioactive compounds of natural origin (nutraceuticals, from “nutrition” and pharmaceuticals”). The observed health benefits of these compounds are of considerable interest to the scientific community, and some have been experimentally tested [104]. This approach is clinically relevant, as many of these natural products act through mechanisms that are similar to those of pharmacological drugs, in the treatment of diabetes and/or insulin resistance, for instance [105,106]. Several therapeutic strategies of nutraceutical containing polyphenols have been used to ameliorate metabolic alterations related to hyperglycemia and T2D. Many of the cited pre-clinical and clinical studies include the following: (i) inhibition of the carbohydrate-digestive enzymes; (ii) modification in the expression or activity of glucose transporters; (iii) inhibition of glycogenolysis; (iv) modulation of the intestinal microbiota by degradation products of bioactive compounds (metabolites) [103], (vi) reduced exposure to high insulin levels, and (vii) consequently alleviated oxidative stress and inflammation.

There are over 8000 different phenolic compounds with various bioactivities, many of these actions likely via the modulation of the NRF2 pathway [107].

Table 1 reports some of the main effects exerted by phenolic compound-based nutraceuticals in both diabetic animal models and patients.

Important results on the effects of phenolic compounds and their underlying mechanisms of action have been obtained through pre-clinical studies. However, data about the specific influence of phenolic compounds on blood insulin concentrations in diabetic animals are still scarce. In one study, in streptozotocin–nicotinamide diabetic rats, a considerable hyperinsulinemic effect of resveratrol was shown [110]. On the other hand, Su and coworkers, using the same model of diabetes, demonstrated that insulinemia was deeply reduced in diabetic rats receiving resveratrol compared with non-treated diabetic rats [108]. Beyond blood insulin concentration, the cellular action of this hormone is extremely important. The data point to the blood glucose-lowering effects of phenolic compounds [111,113,121,130,133,134]. The blood glucose-lowering activity of phenolic compounds may involve both insulin-independent and insulin-dependent effects. In myocardium, isolated from streptozotocin-induced diabetic rats receiving resveratrol (2.5 mg/kg body weight) for 2 weeks, increased phosphorylation of adenosine monophosphate kinase (AMPK), protein kinase B (Akt/PKB), and endothelial nitric oxide synthase (eNOS), was observed, as well as glucose transporter-4 (GLUT4) expression and translocation in an insulin-independent manner compared with diabetic animals not treated with resveratrol [109]. The enhanced GLUT4 expression and insulin receptor (IR) phosphorylation in heart and skeletal muscle of streptozotocin-induced diabetic rats were also confirmed by other study using anthocyanins [129]. Apart from these results, other effects of phenolic compounds have been reported that relate to diabetes. These include increased glycogen synthase activity and reduced glycogen phosphorylase activity in the liver of diabetic rats with improved hepatic glycogen content [110]. Substantially reduced blood triglycerides have also been reported in diabetic rats [111,122,123,132]. In addition, enhanced antioxidant capacity in liver, pancreas [119,123], blood [125], and brain [126] have been observed. It is of importance to note that phenolic compounds also ameliorated diabetic nephropathy [111] and cognitive decline [112,127]. Importantly, phenolic acids and flavonoids lowered levels of interleukin-6 (IL-6) and tumor necrosis factor-alpha (TNF-α) in liver, pancreas [119], and brain [126,127], suggesting their importance in the control of inflammation. These mechanisms played important roles in increasing insulin sensitivity and reversing insulin resistance, ameliorating the diabetes condition and related complications.

Although phenolic compounds have been studied for their potential health benefits, little is known about their metabolic effects in humans. An important issue still much debated related to the anti-diabetic role of phenolic compounds, such as resveratrol, one of the non-flavonoids that has been widely studied in clinical studies, concerns the correct dose and treatment time. It is still uncertain whether low or high doses are more efficient. In this regard, an interesting clinical trial was conducted in 66 patients aged between 20 and 65 years who had T2D, who were supplemented with resveratrol at a dose of 1 g/day for 45 days. This study showed decreased fasting blood glucose, HbA1c, insulin, and insulin resistance, with a concomitant increase in serum HDL-cholesterol levels [133]. On the other hand, resveratrol has been shown to be effective at lower doses as well. In fact, 10 T2D patients received oral resveratrol twice daily (in gelatin capsules containing 5 mg resveratrol), and 9 patients received a placebo (two capsules daily). All nineteen patients were over 18 years of age. After the fourth week, resveratrol significantly decreased fed blood glucose levels and improved insulin sensitivity by decreasing oxidative stress, as shown by urinary ortho-tyrosine level reduction, while it increased the phosphorylation of Akt/PKB in platelets [134]. Interestingly, a meta-analysis comprising a total of 338 adult subjects observed that resveratrol significantly improved glucose control and insulin sensitivity in people with diabetes, but it did not affect glycemic control in non-diabetic people [135], highlighting the need of further studies to better assess the potential benefits of this phenolic compound in humans and the possibility of its potential use as an anti-hyperglycemic agent.

A few clinical trials have also been conducted on the effect of anthocyanins ameliorating insulin resistance under diabetes conditions. Table 1 details a relevant study demonstrating that supplementation with an extract of anthocyanins exerted beneficial metabolic effects in 58 adult subjects with T2D by improving dyslipidemia, enhancing the antioxidant capacity, and preventing insulin resistance [128]. This trial was registered in www.clinicaltrials.gov (accessed on 10 January 2022) as NCT02317211. The total anthocyanin content was 80 mg/capsule, which consisted of 17 different natural purified anthocyanins from bilberry (*Vaccinium myrtillus*) and blackcurrant (*Ribes nigrum*). The relative content of each anthocyanin was as follows: 33.0% of 3-*O-β*-glucosides, 3-*O-β*-galactosides, and 3-*O-β*-arabinosides of cyanidin; 58.0% of 3-*O-β*-glucosides, 3-*O-β*-galactosides, and 3-*O-β*-arabinosides of delphinidin; 2.5% of 3-*O-β*-glucosides, 3-*O-β*-galactosides, and 3-*O-β*-arabinosides of petunidin; 2.5% of 3-*O-β*-glucosides, 3-*O-β*-galactosides, and 3-*O-β*-arabinosides of peonidin; 3.0% of 3-*O-β*-glucosides, 3-*O-β*-galactosides, and 3-*O-β*-arabinosides of malvidin; and 1.0% of 3-*O-β*-rutinoside of cyanidin and delphinidin [136].

The clinical studies that demonstrate the true potential of supplementation with phenolic compounds are far from complete. More research is needed to fully assess the true value of phenolic compound-based nutraceuticals and its utilization in clinical practice, whether as pure compounds, extracts, or even as whole foods. Table 2 shows other important clinical trials that have been or are being conducted to further understand the beneficial effects of phenolic compounds in improving insulin resistance and combating T2D. Considering those studies that have been completed, it was possible to conclude that phenolic compound-based nutraceuticals may be important resources for the prevention and maintenance of vascular health in patients with insulin resistance and T2D with or without associated pharmacological treatment. In addition, the similarity between the beneficial effects of (poly)phenols and pharmacological drugs seems to be linked to several mechanisms of action ranging from inhibition of carbohydrate digestion in the intestine, protection of pancreatic cells and regulation of insulin secretion to increased insulin sensitivity in vital organs. This appears to be through targeting the various specific insulin signal transduction pathways of enzymes/receptors. Since insulin resistance is closely linked to obesity, some of the common mechanisms for the beneficial metabolic effects of phenolic compounds on insulin resistance described in these studies are directed at improving dyslipidemia and inflammation, enhancing the antioxidant capacity, and preventing intestinal dysbiosis that promote constant production of bioactive microbial metabolites. These are key pathways and processes that are involved in the healthy aging process, and therefore, we should greatly benefit from enriching our daily diet with phenolic compounds.

**Figure 4:**
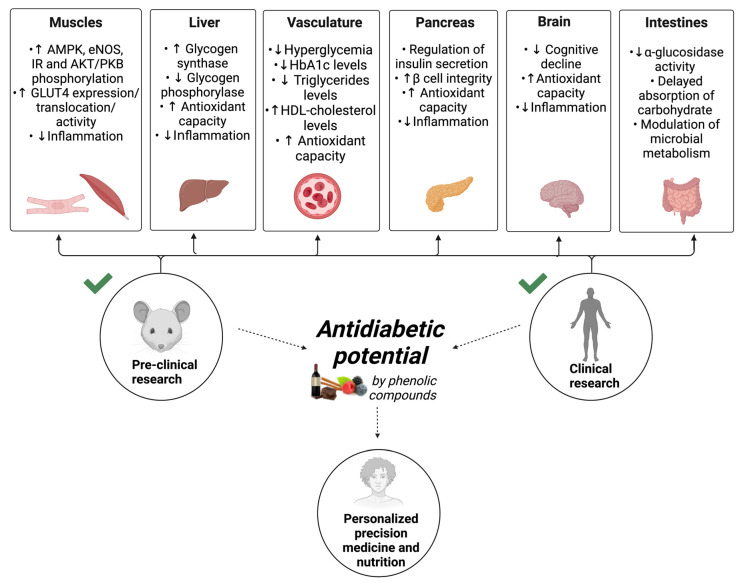
Underlying mechanisms of phenolic compounds against diabetes and its associated complications described herein in pre-clinical and clinical diabetes studies. Future research should focus on personalized studies in precision medicine and nutrition to be applied among persons of different sexes and across different age groups. AMPK: adenosine mono phosphate kinase; AKT/PKB: protein kinase B; eNOS: endothelial nitric oxide synthase; GLUT4: glucose transporter-4; HbA1c: glycosylated hemoglobin; IR: insulin receptor. The arrows point to the final objective of the researches: to prove the antidiabetic potential of phenolic compounds.

Figure 4 synthesizes different mechanisms responsible for the protective activity of phenolic compounds, described herein in pre-clinical and clinical diabetes studies.

## 4. General Summary and Conclusions

The beneficial effects of phenolic compounds in diabetes have been widely investigated using experimental animal models of T1D (e.g., streptozotocin-induced diabetes) and of T2D (e.g., streptozotocin–nicotinamide diabetic rats, fructose-fed streptozotocin-induced diabetic rats, leptin receptor-deficient Zucker diabetic fatty (ZDF) rats, high-fat-diet-fed rats or fructose-fed rats). Of the parameters investigated in these studies, insulin levels require further studies due to contradictory results. Furthermore, phenolic compounds may hold promise as potential therapeutic agents to regulate overall glucose metabolism by reducing oxidative stress-mediated inflammation, cellular apoptosis, and attenuate insulin resistance, as well as ameliorate vascular and metabolic diabetes complications. However, more studies are needed that drive together primary pre-clinical and clinical results with the identification and validation of the potential key health effects of phenolic compounds. This may need to include studies of standardized doses, intake duration, pure compounds versus extracts or even whole food intake, double blinding of the study participants and study staff, and larger numbers of subjects.

Moreover, it should also be of interest to assess whether a combination between some nutraceuticals and some pharmacological drugs might be a more effective method of treatment than single administration alone. Another interesting future direction would be to characterize how the physiological response to nutraceuticals differs among persons of different sexes and across different age groups. Furthermore, an important key issue that should be further studied regarding nutraceuticals is their bioavailability and tissue accumulation after ingestion. The evaluation of possible side effects, if any to report, should also be taken into consideration.

High inter-individual variation in bioavailability is possibly one of the main factors affecting physiological responses in humans, as well as changes in the gut microbiota. To further understand the effect of polyphenol consumption on T2D development and to shed light on the interconnection between the gut microbiome, bioavailability, and bio efficacy, future acute and chronic studies linking these factors with clinical outcomes are not only needed but are highly recommended.

Understanding specific target populations and therapeutic protocols for single, combined, or drug-associated nutraceuticals is also fundamental. Future research should focus on clinical trials to answer some of the important recurrent questions already described. Research questions can be addressed with the use of precision medicine and/or nutrition, investigating the physiology behind nutraceutical mechanisms of action of each phenolic compound. In addition, better patient acceptance of treatment with a natural substance can represent an excellent strategy to prevent and reduce the incidence of cardiovascular risk factors, such as T2D. Thus, considering the use of nutraceuticals as an adjunct to lifestyle changes and pharmacological therapies is strongly recommended to improve patient outcome. Finally, improvements in metabolic health at the global stage cannot be achieved without education and healthy literacy across the population, especially in those most vulnerable and those at high risk of disease development.

## Figures and Tables

**Table 1:** A summary of individual phenolic compounds or extracts possessing antidiabetic effects in pre-clinical and clinical studies.

Phenolic Compounds Class	Compounds	Role in Diabetic Complications	Animal Models/ Diabetic Patients	Dose/Time	References
Stilbenes	Resveratrol	Reduced fed blood glucose levels, insulin concentration, plasma triglycerides	Streptozotocin– nicotinamide diabetic rats	0.5 mg/kg body weight; 14 days	[108]
		Increased phosphorylation of AMPK, eNOS and AKT/PKB, expression of GLUT4 in the myocardium	Streptozotocin- induced diabetic rats	2.5 mg/kg body weight; 2 weeks	[109]
		Decreased fasting blood glucose, HbA1c, increased insulin, improved hepatic glycogen content, increased glycogen synthase activity and reduced glycogen phosphorylase activity	Streptozotocin–nicotinamide diabetic rats	5 mg/kg body weight; 30 days	[110]
		Alleviated diabetic nephropathy by reduced renal dysfunction and oxidative stress	Streptozotocin-induced diabetic rats	5 or 10 mg/kg body weight; 2 weeks	[111]
		Ameliorated cognitive decline by inhibition of hippocampal apoptosis via the Bcl-2/Bax and caspase-3 pathway, improvement of synaptic dysfunction	Streptozotocin-induced diabetic rats	80 mg/kg body weight; 4 weeks	[112]
		Enhanced cerebral vasodilator function, improved cognitive performance	**Patients with T2D**	a single dose of 75 mg	[113]
		Reduced fasting blood glucose, increased serum HDL-cholesterol levels, decreased total-/HDL-cholesterol ratio, increased total antioxidant capacity, decreased plasma MDA levels, upregulation of PPAR-γ and SIRT1 in PBMCs	**Subjects with T2D and coronary heart disease**	500 mg/day; 4 weeks	[114]
		Reduced foot ulcer size and plasma fibrinogen level	**Patients with diabetic foot syndrome**	50 mg/ twice a day; 60 days	[115]
Phenolic acids	Curcumin (Hydroxycinnamic acid)	Improved diabetes-induced endothelial dysfunction through superoxide reduction and PKC inhibition	Streptozotocin-induced diabetic rats	30 and 300 mg/kg body weight; 6 weeks	[116]
		Reduced fasting blood glucose, reduced weight and BMI	**Overweight patients with T2D**	1500 mg/3 times in a day; 10 weeks	[117]
	Ferulic acid (Hydroxycinnamic acid)	Decreased fasting blood glucose levels, reduced level of serum insulin and spleen size. Reduced oxidative stress mediated inflammation and apoptosis	Streptozotocin-induced diabetic rats	50 mg/kg body weight; 8 weeks	[118]
	Gallic acid (Hydrobenzoic acid)	Reduced fasting serum glucose and lipids, improved hepatic and pancreatic antioxidant capacity, lowered levels of IL-6 and TNF-α in liver and pancreas	Fructose-fed streptozotocin-induced diabetic rats	50 mg/kg body weight; 21 days	[119]
Lignans + Phenolic acids	Flaxseed extract (SDG, SECO, LARI, MATA, PINO + ferulic acid, GAE, p-coumaric acid)	Reduced fasting blood glucose, plasma cholesterol, LDL-cholesterol, triglycerides, plasma creatinine, urea and uric acid levels, partially recovers pancreas, liver, and kidney functions	Streptozotocin-induced diabetic rats	0.774 mg/day of lignans + 0.073 mg/day of phenolic acids; 60 days	[120]
Flavonoids	Luteolin (Flavone)	Improved neuronal injury and cognition by attenuating oxidative stress	Streptozotocin-induced diabetic rats	50 and 100 mg/kg body weight; 8 weeks	[121]
	Fisetin (Flavonol)	Diabetic neuropathy modulation by improved motor nerve conduction velocity and reduced inflammation in sciatic nerves by NF-κB inhibition and Nrf2-positive modulation	Streptozotocin-induced diabetic rats	5 and 10 mg/kg body weight; 2 weeks	[122]
	Quercetin (Flavonol)	Reduced pancreatic tissue MDA levels, serum NO concentrations, increased SOD, GSHPx, and CAT enzyme activation in pancreatic homogenates, and preserved pancreatic β-cell integrity	Streptozotocin-induced diabetic rats	15 mg/kg body weight; 4 weeks	[123]
	Total green tea extract (Flavanol)	Reduced fasting blood glucose level, increased total antioxidant capacity and thiol groups in blood	Streptozotocin-induced diabetic rats	3 mg/L through drinking water; 8 weeks	[124]
	Cocoa (Flavanol)	Decreased fasting plasma glucose, HbA1c, and blood pressure levels	**Individuals with T2D and hypertension**	450 mg/day; 8 weeks	[125]
	Hesperidin (Flavanone)	Attenuated streptozotocin-induced neurochemical alterations, increased norepinephrine, dopamine, and serotonin levels, decreased MDA, increased GSH, and decreased IL-6 in brain	Streptozotocin-induced diabetic rats	25, 50 or 100 mg/kg body mass; 21 consecutive days	[126]
	Genistein (Isoflavone)	Reduced hyperglycemia, improved cognition by restoring acetylcholinesterase activity and ameliorated neuroinflammation via decreasing TNF-α, IL-1β, and nitrites in brain	Streptozotocin-induced diabetic mice	2.5, 5 and 10 mg/kg body weight; 30 days	[127]
	Cyanidin, delphinidin, petunidin, peonidin, malvidin extract (Anthocyanidins)	Reduced fasting plasma glucose and HbA1c levels, elevated serum adiponectin and β-hydroxybutyrate concentrations, improved dyslipidemia, enhanced the antioxidant capacity measured in plasma	**Patients with T2D**	160 mg/day; 24 weeks	[128]
	Cyanidin-3-glucoside, delphinidin-3-glucoside, and petunidin-3-glucoside extract (Anthocyanins)	Decreased fed blood glucose, triglycerides levels, enhanced GLUT4 expression and insulin receptor phosphorylation in heart and skeletal muscle, protected pancreatic tissue of apoptosis through regulation of caspase-3, Bax, and Bcl-2 proteins, suppressed MDA levels, and restored SOD and CAT activities in serum	Streptozotocin- induced diabetic rats.	50 mg/kg body weight; 30 days	[129]
	Cyanidin 3-rutinoside, cyanidin 3-glucoside, pelargonidin 3-glucoside and pelargonidin 3-rutinoside extract (Anthocyanins)	Reduced fasting blood glucose, maintain insulin levels and β cell histology	Zucker diabetic fatty rats	125 or 250 mg/kg body weight; 5 weeks	[130]
	Delphinidin and cyanidin extract (Anthocyanidins)	Decreased fasting blood glucose levels and improved glucose tolerance	Hyperglycemic obese mice fed high fat diet	50−500 mg/kg body weight; 16 weeks	[131]
	Cyanidin extract (Anthocyanidins)	Inhibited intestinal α-glucosidase activity and decreased post-prandial glycemic response, delayed absorption of carbohydrates	Diet-induced obese and hyperglycemic mice	50 or 100 mg/kg body weight 60 min prior to an oral gavage of sucrose, starch or glucose	[132]

AMPK: Adenosine mono phosphate kinase; AKT/PKB: Protein kinase B; BMI: body mass index; Bcl-2/Bax: B-cell lymphoma protein 2-associated X; CAT: catalase; eNOS: endothelial nitric oxide synthase; GAE: gallic acid equivalent; GLUT4: glucose transporter-4; GSH: reduced glutathione; GSHPx: glutathione peroxidase; HbA1c: glycosylated hemoglobin; HOMA-IR: homeostatic model assessment of insulin resistance; IL-6: interleukin-6; IL-1β: interleukin-1β; IR: insulin receptor; LARI: lariciresinol; MATA: matairesinol; MDA: malondialdehyde; NF-κB: nuclear factor kappa B; NO: nitric oxide; Nrf2: nuclear erythroid 2-related factor 2; PBMCs: peripheral blood mononuclear cells; PINO: pinoresinol; PPAR-γ: peroxisome proliferator-activated receptor-γ; PKC: protein kinase C; ROS: reactive oxygen species; SDG: secoisolariciresinol diglucoside; SECO: secoisolariciresinol;SIRT1: Sirtuin1; SOD: superoxide dismutase; TNF-α: tumor necrosis factor-alpha; T2D: type 2 diabetes mellitus. In bold are studies performed with diabetic patients.

**Table 2:** Current clinical trials on phenolic compounds as potential therapy for insulin resistance and the diabetic state.

Clinical Trial Identifier No.	Objective	Voluntary and Dose	Recruitment Status	Results (References)
NCT01886989	Investigate the postprandial effects of cocoa supplementation in glucose and lipids, and surrogate markers of atherosclerosis in patients with T2D	18 subjects taking one beverage of cocoa polyphenols (960 mg) reconstituted in water after a high-fat meal challenge	Completed	Cocoa decreased total very LDL and chylomicron particles and increased the concentration of total HDL particles over the 6 h postprandial phase. Serum IL-18 was decreased by cocoa vs. placebo. Polyphenol-rich cocoa lowered dyslipidemia and inflammation following a high-fat dietary challenge in adults with T2D [137].
NCT03049631	Assess the effects of regular consumption of red raspberries (RRB) with and without fructooligosaccharide (FOS) on the composition of the gut microbiota and characterize plasma and urine metabolite profiles	20 insulin resistant and 10 healthy subjects were given 125 g/day of RRB or 125 g/d RRB + 8 g/d FOS for 4 weeks	Completed	Individuals with prediabetes and insulin resistance compared to the reference group: (1) enriched *Blautia obeum* and *Blautia wexlerae* and depleted *Bacteroides dorei* and *Coprococcus eutactus*. *Akkermansia muciniphila* and *Bacteroides* spp. were depleted in the lean PreDM-IR subset; and (2) impaired microbial catabolism of select (poly)phenols resulting in lower 3,8-dihydroxy-urolithin (urolithin A), phenyl-*γ*-valerolactones and phenolic acid concentrations (*p* < 0.05). Controlling for obesity revealed relationships with microbial species that serve as metagenomic markers of diabetes development and therapeutic targets [138].
NCT01766570	Measure the beneficial effects of an optimized berries extracts on diabetes and cardiovascular diseases prevention	60 men and women who were assigned to a 6-week experimental period where they consumed the rich polyphenol berries extract mix (333 mg of polyphenols from strawberries and cranberries)	Completed	Rich polyphenol berries extract mix improved insulin sensitivity in overweight and obese non-diabetic, insulin-resistant human subjects but was not effective in improving other cardiometabolic risk factors [139].
NCT04847999	Verify glucose levels before and after consumption of Ross Chocolates’ blend of sweeteners dark chocolate and conventional chocolate in people with diabetes	Individuals with T1D or T2D (10 participants each) were given a Ross Chocolate or conventional sugar-sweetened dark chocolate bar	Completed	No result posted No publication
NCT02650726	Investigate the effect of purified anthocyanins on high-density lipoprotein and endothelial function in subjects with T2D	80 male and female patients were given daily dose of 320 mg anthocyanin for 24 weeks in a randomized double blinded placebo-controlled trial	Completed	No result posted No publication
NCT01245270	Investigated the acute effect of a standardized bilberry extract on glucose metabolism in T2D	8 male volunteers with T2D were given a single oral capsule of either 0.47 g standardized bilberry extract (36% wet weight anthocyanins) or placebo followed by a polysaccharide drink (equivalent to 75 g glucose) in a double blinded cross over intervention with a 2-week washout period	Completed	The ingestion of a concentrated bilberry extract reduces postprandial glycaemia and insulin in volunteers with T2D. The most likely mechanism for the lower glycemic response involves reduced rates of carbohydrate digestion and/or absorption [140].
NCT01923597	Determine the safety and effect of green tea polyphenols (epigallocatechin gallate) on residual albuminuria of diabetic patients with nephropathy	Patients received four capsules (one capsule = 200 mg of epigallocatechin gallate) of green tea extract per day or placebo for 3 months	Completed	Green tea polyphenols administration reduces albuminuria in diabetic patients receiving the maximum recommended dose of renin-angiotensin (RAS) inhibition. Reduction in podocyte apoptosis by activation of the WNT pathway may have contributed to this effect [141].
NCT02035592	Investigate dose-dependent impact of blueberry powder intake on insulin sensitivity and resistance, cardiovascular disease risk factors, and lung and cognitive function in a population with metabolic syndrome	144 male and female subjects received 26 g of freeze-dried blueberry powder (equivalent to 2 portions of fresh blueberries) per day for 6 months	Completed	A daily intake of 1 cup of blueberries improved endothelial function, systemic arterial stiffness, and attenuated cyclic guanosine monophosphate concentrations. Reduced insulinemia and glucose levels, decreased total cholesterol, and improved HDL-cholesterol, fractions of HDL-particles and Apolipoprotein A1 [142].
NCT04383639	Evaluate the effects of a single intake of a mixture of cocoa and carob (rich in high-molecular-weight polyphenols) in postprandial metabolism in subjects with T2D	The subjects will receive, after overnight fasting, a high-fat, high-sugar breakfast. In treatment A, they will not receive any additional product; in treatment B, they will receive at the same time a mixture of cocoa and carob; in treatment C, they will receive the mixture of cocoa and carob 10 h before breakfast. A total of 6 blood samples will be collected during each visit: 0–30–60–120–180–240–270 min	Recruiting	
NCT02291250	Investigated the acute affect blackcurrants on glucose metabolism in overweight/obese volunteers	16 overweight/obese volunteers will give 200 g of blackcurrants (which contain anthocyanins) or greencurrants (which naturally contain no anthocyanins)	Recruiting	
NCT04419948	Investigate the acute effect of oleocanthal rich extra-virgin olive oil on postprandial hyperglycemia and platelet activation of T2D patients	Non-insulin-dependent diabetic patients will be randomly assigned to consume in five different days white bread (50 g CHO) with butter, butter with ibuprofen, refined olive oil, and olive oil with oleocanthal (250 mg/kg 500 mg/kg)	Recruiting	

(NCT numbers refer to the source of www.clinicalTrails.gov, accessed on 10 January 2022).
